# The tyrosine phosphatase PTPN13/FAP-1 links calpain-2, TBI and tau tyrosine phosphorylation

**DOI:** 10.1038/s41598-017-12236-3

**Published:** 2017-09-18

**Authors:** Yubin Wang, Randy A. Hall, Moses Lee, Andysheh Kamgar-parsi, Xiaoning Bi, Michel Baudry

**Affiliations:** 1 Graduate College of Biomedical Sciences, Western University of Health Sciences, Pomona, CA 91766 USA; 2 College of Osteopathic Medicine of the Pacific, Western University of Health Sciences, Pomona, CA 91766 USA; 3 Emory University School of Medicine, Atlanta, GA 30322 USA

## Abstract

Traumatic brain injury (TBI) increases the risk of Alzheimer’s disease (AD). Calpain activation and tau hyperphosphorylation have been implicated in both TBI and AD. However, the link between calpain and tau phosphorylation has not been fully identified. We recently discovered that the two major calpain isoforms in the brain, calpain-1 and calpain-2, play opposite functions in synaptic plasticity and neuronal survival/death, which may be related to their different C-terminal PDZ binding motifs. Here, we identify the tyrosine phosphatase PTPN13 as a key PDZ binding partner of calpain-2. PTPN13 is cleaved by calpain-2, which inactivates its phosphatase activity and generates stable breakdown products (P13BPs). We also found that PTPN13 dephosphorylates and inhibits c-Abl. Following TBI, calpain-2 activation cleaved PTPN13, activated c-Abl and triggered tau tyrosine phosphorylation. The activation of this pathway was responsible for the accumulation of tau oligomers after TBI, as post-TBI injection of a calpain-2 selective inhibitor inhibited c-Abl activation and tau oligomer accumulation. Thus, the calpain-2-PTPN13-c-Abl pathway provides a direct link between calpain-2 activation and abnormal tau aggregation, which may promote tangle formation and accelerate the development of AD pathology after repeated concussions or TBI. This study suggests that P13BPs could be potential biomarkers to diagnose mTBI or AD.

## Introduction

Tau hyperphosphorylation is a hallmark of several neurological disorders, including Alzheimer’s disease (AD), frontotemporal dementia, traumatic brain injury (TBI) and chronic traumatic encephalopathy (CTE)^1–3^. Many protein kinases and phosphatases have been shown to participate in the regulation of the multiple phosphorylation sites on the tau protein^4,5^. Among them, several non-receptor tyrosine kinases, such as c-Abl, have been identified to regulate tau tyrosine phosphorylation and have been implicated in tau aggregation^6–9^. However, the exact mechanisms relating tau tyrosine phosphorylation to tau pathology have not been elucidated. Calpain activation has also been implicated in many of the same disorders, and calpain-mediated p35 to p25 conversion and the resulting activation of cyclin-dependent kinase 5 (cdk5) was shown to be involved in tau phosphorylation in AD^10–14^, although this hypothesis has been challenged^15^. Other links between calpain and tau hyperphosphorylation involve calpain-mediated truncation of glycogen synthase kinase 3 beta (GSK-3ß)^16,17^, dual specificity tyrosine phosphorylation-regulated kinase 1A (Dyrk1A)^18^ and protein phosphatase 2A (PP2A)^19^. TBI has been shown to be a major risk factor for AD^20,21^, and CTE, resulting from repeated concussions, has been associated with tau hyperphosphorylation^2,22^. However, how TBI results in tau hyperphosphorylation is still not understood.

Over the last few years, our laboratory has focused on understanding the relative roles of two of the major calpain isoforms in the brain, calpain-1 and calpain-2 (aka µ-calpain and m-calpain, respectively) in both physiological and pathological conditions (for review, see^23^). We have shown that these two isoforms are often activated by the same initial stimulus, although with a different time-course of activation and for different durations. Furthermore, we provided evidence that calpain-1 and calpain-2 play opposite functions under both physiological and pathological conditions. Thus, calpain-1 is required for the induction of long-term potentiation (LTP) in hippocampus and for some forms of learning and memory, while calpain-2 activation limits the magnitude of LTP and learning and memory^24,25^. Calpain-1 activation is neuroprotective through the stimulation of survival cascades but calpain-2 activation is neurodegenerative^26–29^. We postulated that these opposite functions of calpain-1 and calpain-2 were the result of the existence of different PDZ domain binding motifs in the C-terminus of calpain-1 and calpain-2^23^. PDZ domains are conserved protein-protein interaction modules within multivalent scaffold proteins^30^, and thus the differential association of these 2 calpain isoforms with distinct PDZ domains could alter the activity/function of these isoforms by localizing them within different clusters of signaling proteins. In particular, calpain-1 exhibits a type II PDZ binding motif, TMFA, across the majority of vertebrates, while calpain-2 exhibits a typical type I PDZ binding domain, FSVL. In this study, we identified a key PDZ binding partner of calpain-2 to be the tyrosine phosphatase, PTPN13, aka FAP-1, which was previously shown to be involved in cell apoptosis^31,32^. We found that calpain-2 cleaves PTPN13, resulting in the inactivation of its phosphatase activity, and the generation of stable breakdown products recognized by PTPN13 antibodies (P13BPs). We also found that PTPN13 regulates the phosphorylation/activation of the protein kinase c-Abl, which was previously shown to phosphorylate tau at tyrosine 394^7,9^. Following TBI, calpain-2 cleaves PTPN13, activates c-Abl, and stimulates tau tyrosine phosphorylation and tau oligomer formation. Moreover, increased levels of P13BPs were found in postmortem brains of AD patients. Thus, calpain-2-mediated truncation of PTPN13 and the resulting c-Abl activation provide a link between TBI and tau phosphorylation, which could account for the increased risk of AD following TBI.

## Results

### Identification of PTPN13 as a calpain-2 PDZ binding partner

**Figure 1:**
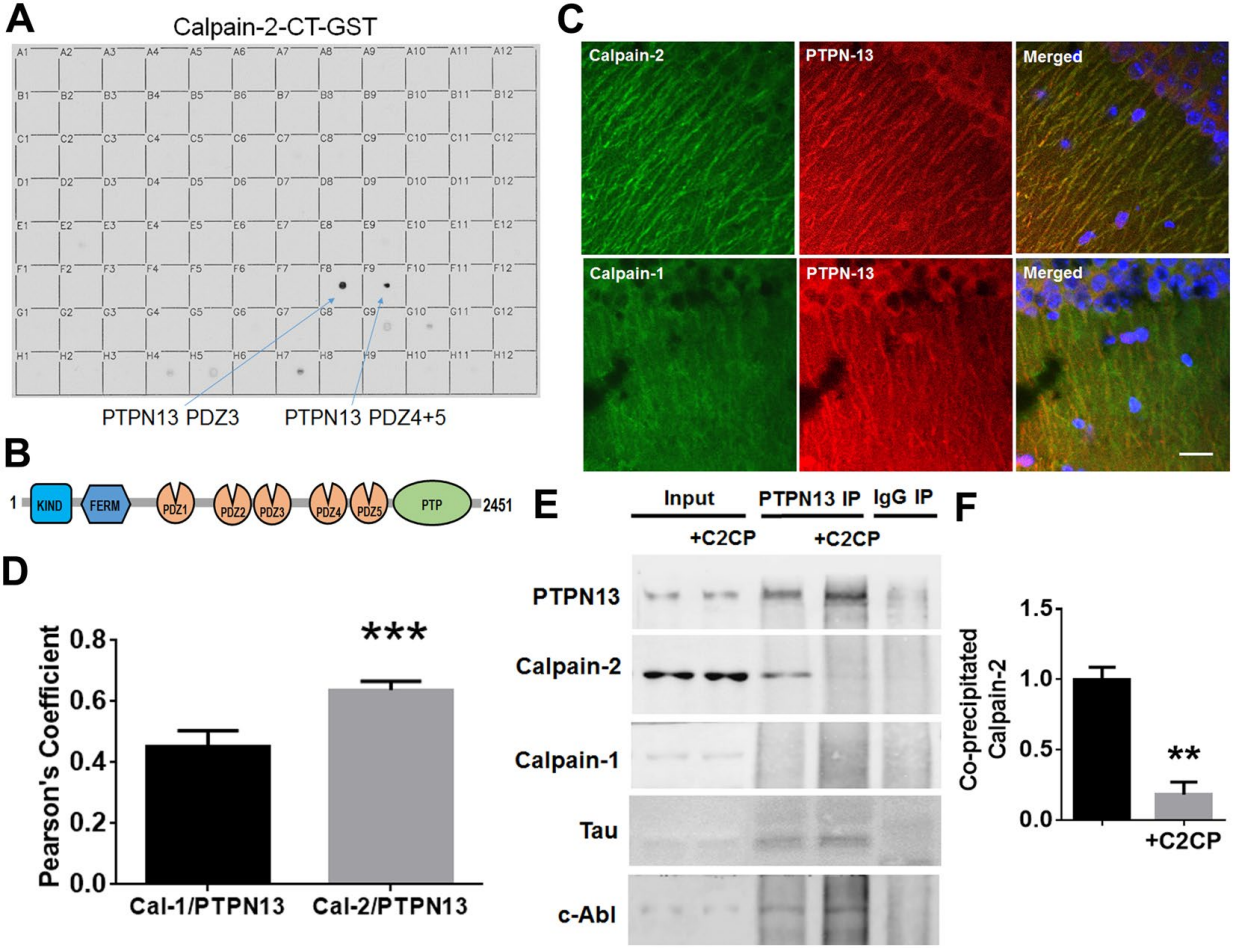
The C-terminus of calpain-2 binds to the PDZ domains of PTPN13. **(A)** Overlay of a PDZ domain array with the GST-tagged C-terminus of calpain-2 (800 nM). The GST-calpain-2 C-terminus exhibited specific binding to several PDZ domains, with PDZ domains 3, 4 and 5 of PTPN13 showing the strongest interactions (arrows). This overlay was repeated three times with similar results. (**B**) Schematic illustration of PTPN13 domains, including kinase non-catalytic C-lobe (KIND) domain and Four-point-one, ezrin, radixin, moesin (FERM) domain at the N-terminus, followed by 5 PDZ domains, and a Protein tyrosine phosphatase (PTP) domain at the C-terminus. (**C**) Co-immunostaining of calpain-2 and PTPN13 or calpain-1 and PTPN13 in the dendrites of CA1 pyramidal neurons. Scale bar = 20 µm. (**D**) Quantification of immunostaining results. Calpain-2 and PTPN13 IMF intensity were analyzed and exhibited a higher Pearson’s correlation coefficient, as compared to calpain-1 and PTPN13 IMF in CA1. N = 4 (animals). In each animal, 4–6 CA1 regions were assessed. ***P < 0.001. Two-tailed *t*-test. (**E**) Co-immunoprecipitation of PTPN13 with calpain-2, tau and c-Abl in P2 homogenates of mouse brain. Calpain-2 C-terminal peptide (C2CP) was added to the homogenates at a final concentration of 10 µM and incubated for 1 h before co-IP. Immunoprecipitation with rabbit IgG was performed as a negative control. Full-length blots are presented in Supplementary Figure 2. (**F**) Quantification of Western blots. Application of calpain-2 C-terminal peptide significantly reduced co-IP of PTPN13 with calpain-2. Data are means ± SEM of 3 independent experiments. **p < 0.01. Two-tailed *t*-test.

We prepared the C-termini (CT) of calpain-1 and calpain-2 as GST fusion proteins and overlaid them on a PDZ domain array, which has been previously described^33,34^. No significant binding to any of the 96 PDZ domains on the array was detected for control GST (Fig. S1A) or GST-calpain-1-CT (Fig. S1B). In contrast, several hits were observed for GST-calpain-2-CT (Figs 1A and S1C). The strongest interactions of the GST-calpain-2-CT fusion protein were with PDZ domains 3, 4 and 5 of the tyrosine phosphatase, PTPN13, aka FAP-1 (Fig. 1A,B).

We next performed a series of experiments to assess whether calpain-2 and PTPN13 were associated in neurons and in brain. Brain sections were dually stained with antibodies against calpain-2 and PTPN13 or calpain-1 and PTPN13 (Fig. 1C). High magnification of cell bodies and dendrites of pyramidal neurons in field CA1 illustrated that calpain-2 and PTPN13 co-localize in dendrites of pyramidal neurons to a much larger degree than calpain-1 and PTPN13. Quantification of the extent of double localization confirmed that calpain-2 was significantly co-localized with PTPN13, relative to calpain-1 (Fig. 1D).

In parallel biochemical studies, we immunoprecipitated PTPN13 from brain homogenates with an antibody against PTPN13 under non-denaturing conditions, in order to pull down proteins that might be associated with PTPN13. As shown in Fig. 1D, calpain-2 but not calpain-1 was clearly co-immunoprecipitated with PTPN13 (Fig. 1E). To shed light on the structural determinants of the interaction, we assessed whether a calpain-2 C-terminal peptide could compete with calpain-2 for binding to PTPN13 in brain homogenates. As shown in Fig. 1E and F, the C-terminal calpain-2 peptide dramatically disrupted calpain-2 co-immunoprecipitation with PTPN13, an observation consistent with the aforementioned direct binding of GST-calpain-2-CT to the PDZ domains of PTPN13 on the PDZ array. These data support the idea that the C-terminus of calpain-2 interacts in native brain tissue with one or several of the PDZ domains of PTPN13.

### PTPN13 is a calpain-2 selective substrate

**Figure 2:**
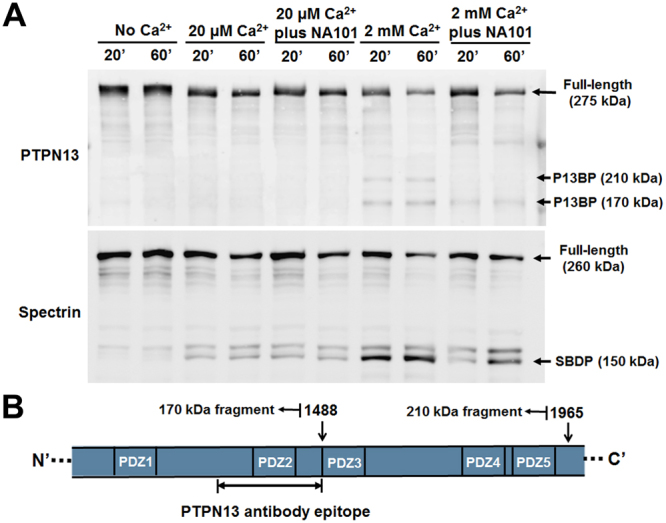
Calpain-2 truncates PTPN13, generating stable breakdown products. (**A**) P2 fractions prepared from mouse brain homogenates were treated with 20 µM or 2 mM Ca^2+^ and incubated for 20 or 60 min at 37 °C. In some groups, a calpain-2 selective inhibitor NA101 (200 nM) was added 10 min before Ca^2+^ treatment. The truncation of PTPN13 and spectrin was analyzed using WB. Full-length blots are presented in Supplementary Figure 2. (**B**) Illustration of the two potential calpain cleavage sites on PTPN13 identified by calpain cleavage site predictor (SVM RBF prediction model). The cleavage at 1488 is predicted to generate a 170 kDa N-terminal fragment and the cleavage at 1965 to generate a 210 kDa N-terminal fragment. These two fragments contain the epitope of PTPN13 antibody (aa1279–1883, as indicated in the antibody manual) used in the WB of panel A and should be detected by this antibody. According to our result, the actual epitope of this antibody should be within aa1279–1488.

Given the proteolytic activity of calpain-2 and its robust binding to PTPN13, we assessed whether PTPN13 might be a substrate for calpain-2. Brain homogenates were incubated with concentrations of calcium activating either endogenous calpain-1 (low calcium concentration, 20 µM) or both calpain-1 and calpain-2 (high calcium concentration, 2 mM). After incubation, samples were immunoblotted with an antibody against PTPN13, as well as an antibody against spectrin (a known substrate for both calpain-1 and calpain-2^35^). As shown in Fig. 2A, incubation with high but not low calcium concentrations resulted in PTPN13 cleavage and the appearance of two breakdown products with molecular weights of 210 and 170 kDa; this effect was significantly reduced by pre-treatment with a selective calpain-2 inhibitor, Z-Leu-Abu-CONH-CH_2_-C_6_H_3_ (3, 5-(OMe)_2_)^24–26,28^, referred to as NA101. We previously reported that NA101 has a Ki of 25 nM against purified calpain-2 versus a Ki of 1.3 µM against calpain-1, indicating that it has a 50-fold selectivity for calpain-2 over calpain-1^26^. In contrast, spectrin was cleaved following incubation with both low and high calcium concentrations (Fig. 2A). These results suggest that PTPN13 is a relatively selective substrate for calpain-2.

Using calpain cleavage site prediction algorithms, we identified two potential cleavage sites in the C-terminus of PTPN13: Asn1488-Thr1489 and Thr1965-Ser1966 (Fig. 2B). Cleavage at 1488 and 1965 would generate breakdown products with molecular weights of 170 and 210 kDa, respectively (Fig. 2B), and both fragments would contain the epitope recognized by the PTPN13 antibody used in the experiments illustrated in Fig. 2A. Thus, the two breakdown products shown in Fig. 2A are likely generated by calpain-2-mediated cleavage of PTPN13 at positions 1488 and 1965. Interestingly, the site at position 1488 is close to PDZ3 and the site at position 1965 close to PDZ5 of PTPN13, suggesting that the predicted cleavage sites are located in close proximity to sites where calpain-2 can bind to PTPN13 (Figs 1A and 2B).

### Calpain-2 activation increases tau tyrosine phosphorylation through a PTPN13/c-Abl pathway

**Figure 3:**
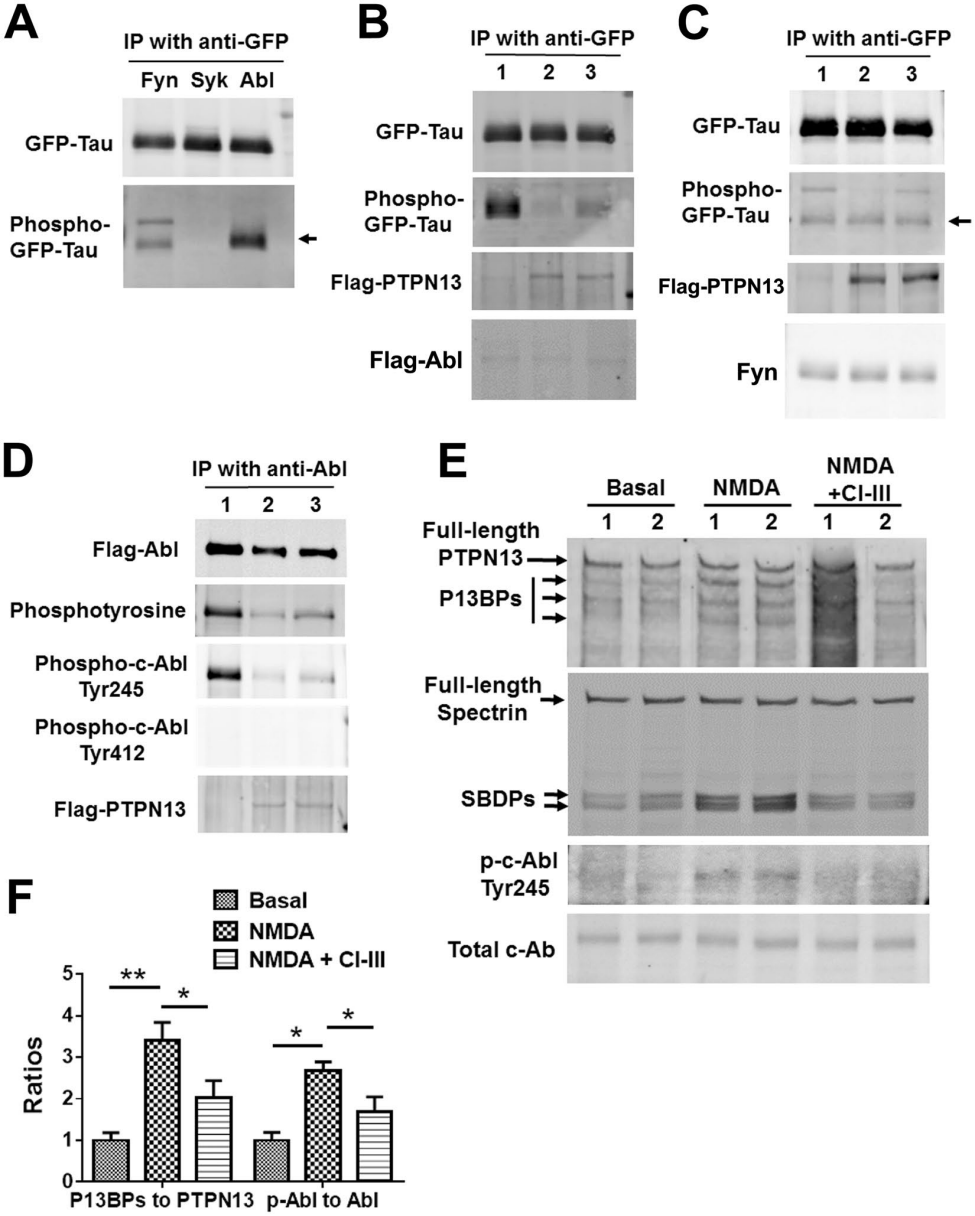
Calpain-2 mediated cleavage of PTPN13 regulates tyrosine phosphorylation of c-Abl and tau. (**A**) Cos-1 cells were transfected with GFP-Tau and c-Fyn (lane 1), GFP-Tau and Flag-SYK (lane 2) or GFP-Tau plus Flag-His-c-Abl (lane 3). GFP-Tau was immunoprecipitated using anti-GFP antibody. Tyrosine phosphorylation of GFP-Tau was examined using anti-phosphotyrosine antibody. Full-length blots are presented in Supplementary Figure 2. (**B**) Cos-1 cells were transfected with GFP-Tau plus Flag-His-Abl (lane 1), GFP-Tau plus Flag-His-Abl plus Flag-PTPN13 WT (lane 2) and GFP-Tau plus Flag-His-Abl plus Flag-PTPN13 D2359A (lane 3). Flag-PTPN13 D2359A is an inactive mutant of PTPN13. GFP-Tau was immunoprecipitated by anti-GFP antibody and was precipitated GFP-Tau was analyzed with an anti-phosphotyrosine antibody. Full-length blots are presented in Supplementary Figure 2. (**C**) Cos-1 cells were transfected with GFP-Tau plus c-Fyn (lane 1), GFP-Tau plus c-Fyn plus Flag-PTPN13 WT (lane 2) and GFP-Tau plus c-Fyn plus Flag-PTPN13 D2359A (lane 3). Tyrosine phosphorylation of GFP-Tau was examined. Full-length blots are presented in Supplementary Figure 2. (**D**) Cos-1 cells were transfected with Flag-c-Abl (lane 1), Flag-c-Abl plus Flag-PTPN13 WT (lane 2) and Flag-c-Abl plus Flag-PTPN13 D2359A (lane 3). Flag-c-Abl was pulled-down using anti-Abl antibody. Tyrosine phosphorylation of Abl was examined using anti-phosphotyrosine, phospho-c-Abl Tyr245 and phospho-c-Abl Tyr412 antibodies. Full-length blots are presented in Supplementary Figure 2. (**E**) NMDA treatment of acute hippocampal slices from calpain-1 KO mice at postnatal day 10. Slices were treated with 10 µM NMDA at 37 °C for 30 min. In one group, slices were pre-treated with a calpain inhibitor (calpain inhibitor III, 10 µM) for 10 min before adding NMDA. Levels of indicated proteins in the collected slices were analyzed by WB. Lane 1 and 2 are two replicates. Full-length blots are presented in Supplementary Figure 2. (**F**) Quantification of WB. Ratios of P13BPs to full-length PTPN13 and phospho-c-Abl Tyr245 to total c-Abl were significantly increased with NMDA treatment. Treatment with CI-III significantly inhibited NMDA-mediated changes. Data are means ± SEM of 4 independent experiments. *p < 0.05, **p < 0.01. Two-way ANOVA followed by Bonferroni test.

Three different tyrosine kinases have been implicated in tau tyrosine phosphorylation: fyn, syk and c-Abl^4^. We first determined which of these tyrosine kinases might be directly involved in tau tyrosine phosphorylation. COS-1 cells were co-transfected with GFP-tau and each of the kinases separately. As shown in Fig. 3A, only fyn and c-Abl increased tau phosphorylation under these conditions. Since PTPN13 is a tyrosine phosphatase, we assessed whether PTPN13 could dephosphorylate phospho-tyrosine tau after its phosphorylation with c-Abl. For these experiments, we transfected COS-1 cells with GFP-tau, Flag-His-Abl and PTPN13 or a mutated form of PTPN13 (D2359A), which partially inactivates PTPN13. PTPN13 resulted in dephosphorylation of phospho-GFP-tau to a much greater extent than mutated PTPN13 (Fig. 3B). When a similar experiment was done with fyn instead of c-Abl, PTPN13 did not affect phospho-tyrosine tau levels induced by fyn (Fig. 3C).

While these data suggested that PTPN13 could dephosphorylate phospho-tyrosine tau after its phosphorylation with c-Abl, the results could be interpreted differently, as for example it could be the case that PTPN13 dephosphorylates and inactivates phospho-tyrosine-c-Abl, since c-Abl has 2 tyrosine residues, whose phosphorylation is required for kinase activity, tyrosine 245 and tyrosine 412^36^. To test this hypothesis, we determined the effects of PTPN13 on phospho-tyrosine c-Abl using two antibodies specific for c-Abl phosphorylated at Tyr245 or Tyr 412. These experiments revealed that PTPN13 could dephosphorylate c-Abl phosphorylated at Tyr 245, although the lack of c-Abl phosphorylated at tyr 412 prevented determination as to whether PTPN13 could also dephosphorylate phospho-tyr 412 c-Abl (Fig. 3D).

In addition, co-immunoprecipitation showed that tau, c-Abl and calpain-2 associated with PTPN13 in mouse brain homogenates (Fig. 1E). The calpain-2 C-terminal peptide did not inhibit the association of tau and c-Abl with PTPN13 (Fig. 1E), indicating that the interaction of tau and c-Abl with PTPN13 does not rely on calpain-2 C-terminal domain.

While the above results indicated that calpain-2 could cleave PTPN13 and that PTPN13 could dephosphorylate c-Abl at tyrosine 245, they did not determine whether calpain-2-mediated cleavage of PTPN13 resulted in its inactivation and increased tyrosine phosphorylation of c-Abl at tyrosine 245. To address this question, we incubated acute hippocampal slices prepared from neonatal calpain-1 knock-out mice with NMDA and determined the effects of calpain-2 activation on spectrin degradation, PTPN13 levels, and c-Abl tyrosine phosphorylation at tyrosine 245 (Fig. 3E,F). We previously used NMDA treatment of neonatal hippocampal slices as a tool to activate endogenous calpain and the use of calpain-1 knock-out mice provided for the selective activation of calpain-2 by NMDA treatment^26^. Under these conditions, calpain-2 was activated, as spectrin was cleaved to produce the typical spectrin breakdown products (SBDPs). Moreover, PTPN13 was cleaved, generating several breakdown products, and c-Abl was phosphorylated at tyrosine 245. These effects were significantly blocked when slices were incubated in the presence of a calpain inhibitor (calpain inhibitor III). These results reveal that calpain-2 activation results in PTPN13 cleavage and inactivation, leading to increased c-Abl phosphorylation at tyrosine 245, a phosphorylation event that is required for c-Abl activity^36^.

### Enhanced PTPN13 cleavage and c-Abl activation following TBI

**Figure 4:**
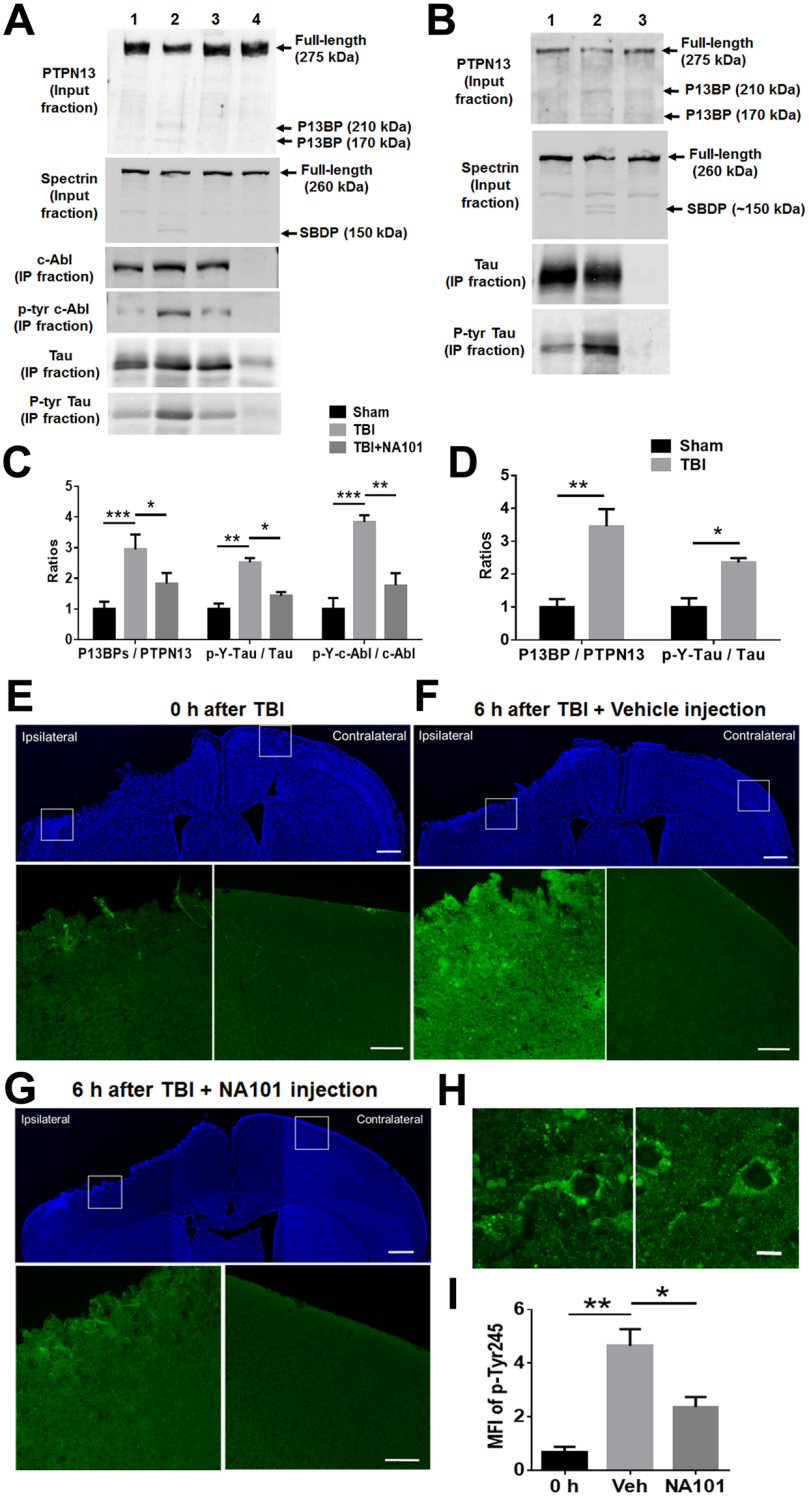
TBI triggers calpain-2 mediated PTPN13 cleavage and tyrosine phosphorylation of c-Abl and tau. (**A**) A calpain-2 selective inhibitor inhibits TBI-triggered PTPN13 cleavage and tyrosine phosphorylation of c-Abl and tau. Ipsilateral cortex of WT mice was collected and homogenized 6 h after surgery. C-Abl or tau was then immunoprecipitated from homogenates to test its tyrosine phosphorylation. *Lane* 1, Sham surgery. Immunoprecipitation was performed with tau or c-Abl antibody. *Lane* 2, Controlled cortical impact (CCI). Immunoprecipitation was performed with tau or c-Abl antibody. *Lane* 3, NA101 was injected i.p. 1 h after CCI. Immunoprecipitation was performed with tau or c-Abl antibody. *Lane* 4, Sham surgery. Immunoprecipitation was performed with mouse or rabbit IgG as a negative control. Full-length blots are presented in Supplementary Figure 2. (**B**) Ipsilateral cortex of calpain-1 KO mice was collected and homogenized 6 h after CCI or sham surgery. PTPN13 and spectrin cleavage and tau tyrosine phosphorylation were analyzed with WB. *Lane* 1, Sham surgery. Immunoprecipitation was performed with tau antibody. *Lane* 2, CCI. Immunoprecipitation was performed with tau antibody. *Lane* 3, Sham surgery. Immunoprecipitation was performed with mouse IgG as a negative control. Full-length blots are presented in Supplementary Figure 2. (**C**) Quantification of WB results similar to panel A. The ratios of P13BPs to PTPN13, phospho-tyrosine tau to total tau, and phospho-Tyr245 of c-Abl to total c-Abl were significantly increased following CCI. Injection with NA101 significantly inhibited those changes following CCI. Data are means ± SEM of 3–5 independent experiments. *p < 0.05, **p < 0.01, ***p < 0.001. Two-way ANOVA followed by Bonferroni test. (**D**) Quantification of WB results similar to panel B. The ratios of P13BPs to PTPN13, and phospho-tyrosine tau to total tau were significantly increased following CCI. Data are means ± SEM of 3 independent experiments. *p < 0.05, **p < 0.01. Two-way ANOVA followed by Bonferroni test. (**E**–**G**) IHC with a phospho-c-Abl (Tyr245) antibody in coronal brain sections (Bregma −0.58 mm) 6 h after TBI. Vehicle (5% DMSO in PBS) or NA101 (0.3 mg/kg) was injected i.p. 1 h after TBI. Scale bar in the DAPI images under low-magnification, 500 µm. Scale bar in the zoomed-in images, 100 µm. (**H**) High-magnification images of phospho-c-Abl (Tyr245) staining of cortical neurons in ipsilateral cortex 6 h after TBI. Scale bar, 10 µm. (**I**) Quantification of IHC results. Three coronal brain sections (Bregma −0.58 mm, −1.58 mm and −1.94 mm) from each brain were immunostained and IMF intensity analyzed. Changes in phospho-Tyr245 level were calculated as the mean fluorescence intensity (MFI) of p-Tyr245 staining in the ipsilateral side of the insult subtracted by the MFI of p-Tyr245 staining in the contralateral side. Changes in p-Tyr245 in all 3 sections from the same brain were averaged. N = 3–5 (animals). *p < 0.05, **p < 0.01. One-way ANOVA followed by Bonferroni test.

We next tested the hypothesis that calpain-2 mediated truncation of PTPN13 might take place after TBI, thus providing a potential link between calpain-2 activation and tau hyperphosphorylation, which has been shown to be a consequence of TBI and concussion^2,3^. We recently reported that in the controlled cortical impact model of TBI in wild-type mice calpain-2 activation was delayed and prolonged and responsible for producing cell death^37^. We first determined the levels of full-length PTPN13, P13BPs, c-Abl, phospho-Tyr245 c-Abl, tau and phospho-tyrosine tau in WT mice after TBI using Western Blot (WB) (Fig. 4A,B). P13BPs (170 and 210 kDa), which were identical to those found after calpain-2-mediated PTPN13 truncation, were detected in the ipsilateral cortical tissue 6 h after TBI. Spectrin truncation was detected as well after TBI. In parallel, increased levels of c-Abl and tau tyrosine phosphorylation were observed. Intraperitoneal (i.p.) injection of NA101 (0.3 mg/kg) 1 h after TBI significantly blocked PTPN13 and spectrin truncation and the increase in c-Abl and tau tyrosine phosphorylation (Fig. 4A,C), suggesting that calpain-2 activation is required for those changes after TBI.

We then used immunohistochemistry (IHC) to verify whether phosphorylation of c-Abl at tyrosine 245 was increased under these conditions. Levels of phospho-Tyr245 were significantly increased in the area surrounding the impact in the ipsilateral cortex of WT mice 6 h after TBI, as compared to 0 h (Fig. 4E,F,H and I). Treatment with NA101 1 h after TBI markedly reduced TBI-induced increase in tyrosine phospho-c-Abl levels 6 h after TBI (Fig. 4G and I).

To verify that PTPN13 cleavage and tau tyrosine phosphorylation following TBI was mediated by calpain-2 but not calpain-1, we tested the levels of full-length PTPN13, P13BPs, tau and phospho-tyrosine tau in calpain-1 KO mice after TBI using Western Blot (Fig. 4B and D). PTPN13 cleavage and tau tyrosine phosphorylation were significantly increased 6 h after TBI in the ipsilateral cortex of calpain-1 KO mice. The extent of these changes was comparable with that found in WT mice (Fig. 4C vs D), suggesting that calpain-1 activation does not participate in these changes.

###  Calpain-2 and c-Abl activation contribute to tau oligomer accumulation following TBI

**Figure 5:**
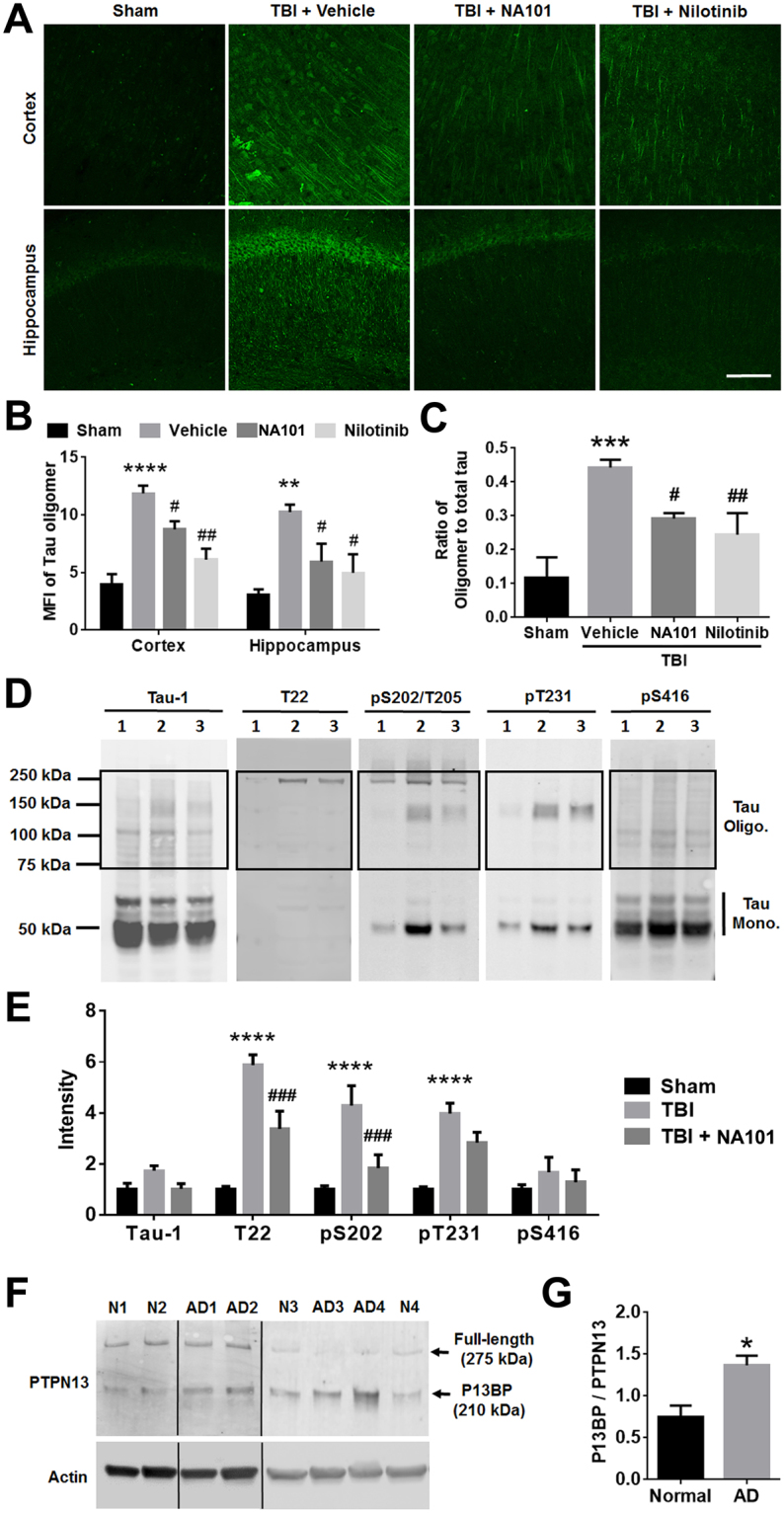
Inhibition of calpain-2 or c-Abl attenuates the formation of tau oligomers in brain following TBI. **(A)** IHC with an anti-tau oligomer antibody (T22) in cortical and hippocampal CA1 areas of coronal brain sections 24 h after TBI or sham surgery. Vehicle (5% DMSO in PBS), NA101 (0.3 mg/kg) or Nilotinib (25 mg/kg) was injected i.p. 1 h after TBI. Scale bar = 100 µm. (**B**) Quantification of IHC results. Three coronal brain sections (Bregma −1.58 mm, −1.94 mm and −2.30 mm) from each brain were imaged. MFIs in cortical and CA1 areas from each section were measured in ImageJ. N = 3 for Sham, C2I and Nilotinib. N = 5 for Vehicle. **p < 0.01, ****p < 0.0001 Sham vs. Vehicle, ^#^p < 0.05 C2I vs. Vehicle, ^##^p < 0.01 Nilotinib vs. Vehicle. Two-way ANOVA followed by Bonferroni test. (**C**) ELISA analysis of brain homogenates using Tau-5 and T22 antibodies 24 h after TBI or sham surgery. Vehicle (5% DMSO in PBS), NA101 (0.3 mg/kg) or Nilotinib (25 mg/kg) was injected i.p. 1 h after TBI. The ratios of tau oligomers to total tau were analyzed. N = 4 (animals). ***p < 0.001 sham vs. vehicle, ^#^p < 0.05 vehicle vs. C2I. ^##^p < 0.01 vehicle vs. Nilotinib. One-way ANOVA followed by Bonferroni test. (**D**) Levels of Tau monomers and oligomers and Serine/Threonine phosphorylation of tau at S202, T205, T231 and S416 in ipsilateral cortical homogenates collected 24 h after sham (lane 1) or TBI (lane 2) or TBI plus NA101 injection (lane 3) were analyzed with WB of non-reducing SDS-PAGE (NuPAGE 4–12% Bis-Tris gel). Full-length blots are presented in Supplementary Figure 2. (**E**) Quantification of tau oligomers (boxed area) as detected by WB. N = 3 (animals). ****p < 0.0001 sham vs. TBI. ^###^p < 0.001 TBI vs. TBI + NA101. Two-way ANOVA followed by Bonferroni test. (**F**) Post-mortem hippocampal samples from 4 AD patients and 4 age-matched controls﻿ (N) were probed with antibodies against PTPN13 and actin in WB. Full-length PTPN13 at 275 kDa and a P13BP at approximately 210 kDa were detected. (**G**) Quantification of the Western blots. The ratio of P13BP to full-length PTPN13 was significantly higher in AD patients than that in controls. N = 4. *p < 0.05. Two-tailed *t*-test.

TBI triggers the rapid accumulation of tau oligomers in the brain as early as 4 h after TBI^38^. Tau oligomers play a critical role in the initiation and spreading of tau pathology^39–42^. To test whether calpain-2 and c-Abl were involved in this process, we injected NA101 or a brain-penetrating c-Abl inhibitor, nilotinib, to WT mice 1 h after TBI, and examined the formation of tau oligomers 24 h after TBI using IHC, ELISA and WB with an antibody that specifically recognizes oligomeric Tau (T22). With IHC, levels of tau oligomers were dramatically increased in the ipsilateral cortex and hippocampus 24 h after TBI (Fig. 5A and B), a result consistent with a previous report in rats^38^. Both NA101 and nilotinib significantly reduced TBI-induced tau oligomer accumulation in cortex and hippocampus (Fig. 5A and B). To verify this result, homogenates of the cortical region adjacent to the lesion were prepared following the same treatments, and the ratios of tau oligomers to total tau in homogenates were examined by ELISA using T22 and Tau-5 antibodies. As expected these ratios were increased following TBI, and the increased were significantly reduced by NA101 or Nilotinib treatment (Fig. 5C). The same homogenates were processed for non-reducing SDS-PAGE and tau oligomers were examined by WB using T22 antibodies. In addition, we also tested the effects of NA101 on the increase in phosphorylation of other sites in the tau protein, tau S202/T205, T231 and S416 using phospho-specific antibodies (Fig. 5D and E). Intensities of several tau oligomer bands detected by T22, as well as by pS202/T205, pT231 and pS416 antibodies were significantly increased after TBI. Increases in levels of T22 and pS202/T205, but not pT231 and pS416 were significantly reduced by NA101 injection 1 h after TBI. The above results suggest that activation of calpain-2 and c-Abl contributes to the accumulation of tau oligomers in the brain following TBI.

Our results suggested the possible involvement of calpain-2-mediated PTPN13 in AD pathology. Thus, we examined whether calpain-2-mediated PTPN13 fragments could be detected in AD brain. Protein samples from hippocampus of 4 post-mortem AD patients and 4 age-matched controls were run on Western blots and labeled with an anti-PTPN13 antibody. A PTPN13 fragment with a molecular weight of approximately 210 kDa was present in hippocampus homogenates from both normal subjects and age-matched AD patients (Fig. 5F). However, the ratio of P13BP over full-length PTPN13 was significantly increased in AD patients, as compared to normal subjects (Fig. 5G).

## Discussion

PTPN13 belongs to the family of protein tyrosine phosphatases (PTP) and has been mostly studied for its role in cancer^32^. PTPN13 is a large protein with a PTP domain toward its C-terminus, five PDZ domains, and a four-point-one, ezrin, radixin, moesin (FERM) domain that binds to plasma membrane and cytoskeletal elements (Fig. 1B). This protein binds and dephosphorylates the Fas receptor, which suggests it participates in apoptosis^31^, but it also regulates the Rho signaling pathway^43^. Little is known regarding potential roles for PTPN13 in the brain or in neurons. Our results indicate that PTPN13 is a key PDZ binding partner for calpain-2 in the brain. This interaction was first observed using an array of PDZ domain-containing proteins, and confirmed by co-localization and co-immunoprecipitation assays. The co-immunoprecipitation assays also confirmed that the interaction between PTPN13 and calpain-2 takes place in the C-terminal domain of calpain-2, which contains a type I PDZ-binding motif. No interaction was found between PTPN13 and calpain-1, which is consistent with the fact that calpain-1 exhibits a type II PDZ-binding motif. Interestingly, calpain-2 cleaves PTPN13 at two locations in its C-terminal region, generating two relatively stable N-terminal breakdown products, which were tentatively identified at Asn1488-Thr1489 and Thr1965-Ser1966. As no stable C-terminal breakdown products predicted to contain the PTP domain were found after cleavage, we postulated that calpain-2-mediated cleavage might inactivate the enzyme’s phosphatase activity. This prediction was verified and we further established c-Abl as a novel downstream substrate of PTPN13. Our data suggested that c-Abl phosphorylation at Tyr245 would increase (and c-Abl activity would therefore be enhanced) following calpain-2-mediated PTPN13 cleavage, and this was confirmed in hippocampal slices. C-Abl has recently been associated with both AD and Parkinson’s disease (PD). In the case of AD, c-Abl has been shown to phosphorylate tau at tyrosine 394, and this site is phosphorylated in human neurofibrillary tangles^9^. In the case of PD, c-Abl phosphorylates and activates parkin, leading to mitochondrial abnormalities and increased oxidative stress^44^.

We recently found that calpain-2 was activated following TBI, and that this activation plays an important role in cell death in the days following TBI^37^. Results presented here demonstrate that PTPN13 is cleaved by calpain-2 following TBI, resulting in c-Abl and tau phosphorylation at tyrosine residues. Systemic injection of a selective calpain-2 inhibitor, NA101, 1 h after TBI at a dose of 0.3 mg/kg, which we previously showed to significantly prevent calpain-2 activation in the brain^25,28^, almost completely eliminated c-Abl activation and increased tyrosine tau phosphorylation. Moreover, injection of NA101 or of a c-Abl inhibitor, nilotinib, significantly reduced the formation of tau oligomers 1 day after TBI, suggesting that the calpain-2 → PTPN13 → c-Abl → tau phosphorylation pathway is involved in this process. Interestingly, NA101 injection significantly reduced the level of pS202/T205 after TBI. Levels of pT231 were partially but not significantly reduced by NA101 injection. In contrast, levels of pS416 were not affected by NA101. GSK-3ß and cdk5 have been shown to directly phosphorylate tau at S202, T205 and T231, while casein kinase 1 (CK1) and protein kinase A (PKA) directly phosphorylate tau at S416^4^. Calpain has been shown to regulate GSK-3ß^45^ and cdk5^13^, and it is therefore possible that calpain-2 activation leads to increased phosphorylation of tau at multiple sites through the regulation of different protein kinases and phosphatases. Recently, accumulating evidence has shown that the propagation of tau oligomers plays a critical role in the development of tau pathology. Injection of tau oligomers derived from AD brain disrupts memory and propagates abnormal aggregation of endogenous tau in WT mice^39–42^. Further studies need to be done to examine the effect of long-term treatment a with calpain-2 inhibitor on neurofibrillary tangle (NFT) formation after TBI. Finally, our results indicate that calpain-2-mediated PTPN13 truncation is enhanced in AD brain, as compared to normal age-matched brain.

It is worth discussing how calpain-2 is activated following TBI. Calpain-2 is located at extrasynaptic sites and activated only when extrasynaptic glutamate level increases, which occurs predominantly under pathological conditions^26^. We proposed that calpain-2 activation elicited by extrasynaptic NMDAR stimulation is the result of the association of NR2B subunit of NMDARs with RasGRF1, which provides a link between extrasynaptic NMDARs and ERK activation^46^. Indeed, we have previously shown that ERK activation directly phosphorylates and activates calpain-2^47^. It has also been repeatedly shown that NR2B subunits are enriched in extrasynaptic NMDARs^48^, and that their activation is critical for excitotoxicity^49^. Thus, this pathway is likely responsible for the prolonged activation of calpain-2 upon stimulation of extrasynaptic NMDA receptors following TBI.

**Figure 6:**
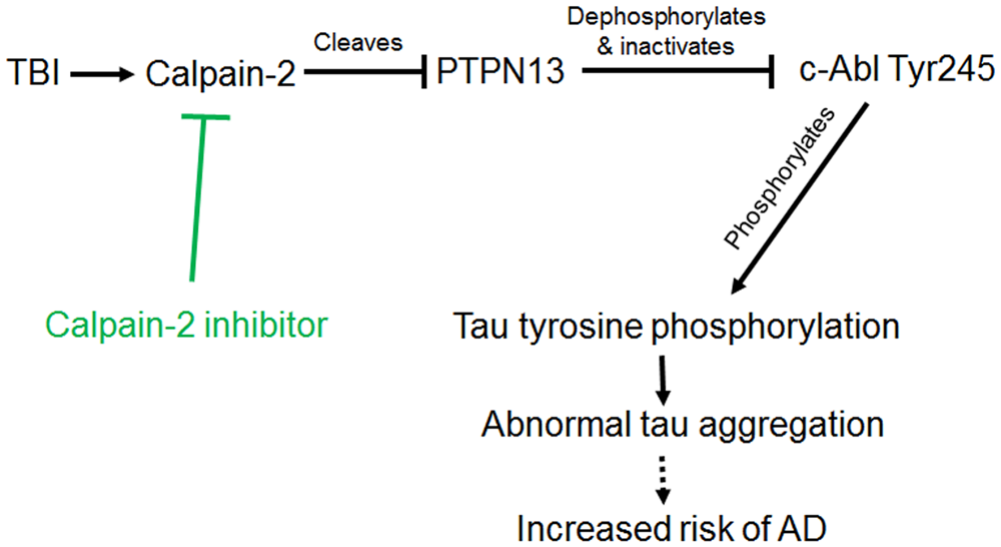
A new link between TBI, calpain-2, tauopathy and AD pathogenesis. TBI triggers calpain-2 activation. PTPN13 is cleaved and inactivated. This results in increased tyrosine phosphorylation of c-Abl at tyr245, which increases its kinase activity and phosphorylates tau at tyr394. Elevation of tau tyrosine phosphorylation contributes to tau oligomer accumulation, which may lead to increased risk of AD or AD-related disease. Administration of a calpain-2 selective inhibitor post-TBI reduces TBI-induced calpain-2 activation, tau tyrosine phosphorylation and tau oligomer formation, thus may inhibit tauopathy and AD pathogenesis.

All these results are summarized in a schematic diagram shown in Fig. 6. We postulate that calpain-2 activation following TBI (and possibly concussion) could lead to PTPN13 truncation, resulting in increased phosphorylation of c-Abl and its activation. In turn, c-Abl activation could lead to tau tyrosine phosphorylation and abnormal tau aggregation. It is possible that c-Abl activation also induces parkin phosphorylation and mitochondrial abnormalities. Importantly, TBI is a risk factor for both AD and PD, and it will be interesting to determine whether parkin is abnormally phosphorylated following TBI in various brain structures. It is also interesting to note that the hallmark of chronic traumatic encephalopathy (CTE) is a massive increase in tau phosphorylation^50^. While there is currently no evidence that tau is phosphorylated at tyrosine 396 in CTE brain, it is possible that repeated concussions activate the calpain-2 → PTPN13 → c-Abl → tau phosphorylation pathway, leading to NFT formation. Furthermore, as PTPN13 has previously been shown to participate in apoptosis, it is possible that calpain-2 mediated truncation/inactivation of PTPN13 is also involved in neuronal apoptosis under various pathological conditions.

It was previously shown that blood levels of a marker of calpain activation, an N-terminal fragment of spectrin generated by calpain, were correlated with the pathological outcomes of mild TBI (mTBI)^51^, and it is therefore likely that the pathway described here is also activated by mTBI. Additionally, it is possible that the P13BP(s) identified here could be further characterized and could potentially serve as novel blood biomarkers for the diagnosis of mTBI or AD. Finally, our results suggest that a selective calpain-2 inhibitor might be beneficial not only for the treatment of TBI, but also for preventing the development of CTE following repeated concussions, as well as for more chronic neurodegenerative disorders such as AD and PD.

## Methods

### Animals

Animal use in all experiments followed NIH guidelines and all protocols were approved by the Institution Animal Care and Use Committee of Western University of Health Sciences. Calpain-1 KO mice on a C57Bl/6 background were obtained from a breeding colony established from breeding pairs generously provided by Dr. Chishti (Tufts University). C57Bl/6 mice were purchased from Jackson Labs and were the corresponding wild-type (WT).

### Materials

#### DNA Constructs

Flag-PTPN13 were generously provided by Dr. Carl-Henrik Heldin (Uppsala University, Sweden). Flag-PTPN13 D2359A was constructed based on Flag-PTPN13 using the QuikChange II Site-Directed Mutagenesis Kit (Agilent Technologies). EGFP-Tau (WT human tau containing human four-repeat tau lacking the N-terminal sequences (4R0N) and contains exons 1, 4 and 5, 7, and 9–13, intron 13, and exon 14)(plasmid #46904), c-Fyn (human) (plasmid #16032), Flag-SYK (human)(plasmid #20646) and Flag-His-c-Abl (human) (plasmid #52684) were purchased from Addgene. GST-calpain-1 C-terminus and GST-calpain-2 C-terminus expression plasmids were constructed on pGEX-3x vector.

#### Antibodies

The primary antibodies used for WB were PTPN13 (1:1000, LS-C148268, LifeSpan BioSciences), calpain-1 (1:800, 2556, CST), calpain-2 (1:1000, LS-B12657, LifeSpan BioSciences), tau-5 (1:2000, AHB0042, Thermo Fisher Scientific), tau-1 (1:2000, MAB3420, EMD Millipore), anti-Tau oligomer T22 (1:300, ABN454, EMD Millipore), phospho-tau Ser202/Thr205 AT8 (1:1000, MN1020, Thermo Fisher), phospho-Tau Thr231 AT180 (1:1000, MN1040, Thermo Fisher), phospho-tau Ser416 (1:1000, p1573–416, PhosphoSolutions), c-Abl (1:1000, 2862, CST), spectrin (1:1000, MAB1622, EMD Millipore), phosphotyrosine (05–321, EMD Millipore), Phospho-c-Abl Tyr245 (1:800, 2861, CST), Phospho-c-Abl Tyr412 (1:800, 2865, CST) and actin (1:10000, A2228, Sigma-Aldrich). The secondary antibodies used for WB were IRDye 680RD goat anti-rabbit (1:10000, 926–68071, LI-COR) and IRDye 800CW goat anti-mouse (1:10000, 926–32210, LI-COR).

#### Peptide

The calpain-2 C-terminal peptide was synthesized by ABI Scientific. The sequence is TIQLNLASWLSFSVL (mouse calpain-2 C-terminus).

### Overlay of PDZ array

Fusion proteins were purified and overlays of the PDZ domain array were performed as previously described^33,34^. Briefly, 1 μg of hexahistidine-tagged PDZ domain fusion proteins were spotted onto nitrocellulose, dried overnight, and then overlaid with GST-alone (control), GST-calpain-1-CT, or GST-calpain-2-CT (800 nM). Membranes were washed three times and incubated with an HRP-coupled anti-GST monoclonal antibody (Amersham Pharmacia Biotech) and binding of calpain-1-CT or calpain-2-CT fusion protein was visualized using enhanced chemiluminescence.

### Calpain cleavage site prediction

Prediction of calpain cleavage site(s) in PTPN13 was performed using the online cleavage site predictor (http://calpain.org/predict.rb?cls=substrate). SVM RBF prediction model was used. The query sequence (1000 aa maximum) was aa1001–1999 of PTPN13 [Mus musculus] (NP_035334.2). This region was selected because it includes the epitope of PTPN13 antibody LS-C148268 which is aa1279–1883. The top three predicted cleavage sites in this region were 1488, 1023 and 1965. The molecular weights (MWs) of the fragments generated by the cleavage at 1488 are 170 kDa (N-terminal) and 105 kDa (C-terminal). The MWs of the fragments generated by 1023 are 125 kDa (N-terminal) and 150 kDa (C-terminal). The MWs of the fragments generated by 1965 are 210 kDa (N-terminal) and 65 kDa (C-terminal). Among them, the 170 kDa fragment generated by cleavage at 1488 and 210 kDa generated by cleavage at 1965 should be recognized by PTPN13 antibody and fit the MWs of the breakdown products detected in WB (Fig. 2A)

### Cell lines and transfection

Cos-1 cells (ATCC) were grown in DMEM supplemented with 10% (v/v) fetal bovine serum (FBS) (Invitrogen). For transient expression, cells were transfected with the respective DNA constructs by Lipofectamine 2000 (Invitrogen).

### Controlled cortical impact

CCI model was established in mice following the protocol described in previous publications^52–55^. Mice (3-month old, 25–30 g) were anesthetized using isoflurane and fixed in a stereotaxic frame with a gas anesthesia mask. A heating pad was placed beneath the body to maintain body temperature around 33–35 °C. The head was positioned in the horizontal plane. The top of the skull was exposed, and a 5-mm craniotomy was made using a micro drill lateral to the sagittal suture, and centered between Bregma and Lambda. The skull at the craniotomy site was carefully removed without damaging the dura. The exposed cortex was hit by a pneumatically controlled impactor device (AMS-201, Amscien). The impactor tip diameter was 3 mm, the impact velocity was 3 m/sec, and the depth of cortical deformation was 0.5 mm. After the impact, the injured region was sutured using tissue adhesive (3 M) and the mice were placed in a 37 °C incubator until they recovered from anesthesia. For sham surgery, mice were sutured after craniotomy was performed.

### Immunohistochemistry

Mice were anesthetized and perfused intracardially with freshly prepared 4% paraformaldehyde in 0.1 M phosphate buffer (PB, pH 7.4). After perfusion, brains were removed and immersed in 4% paraformaldehyde at 4 °C for post-fixation, then in 15% and 30% sucrose at 4 °C for cryoprotection. Coronal frozen sections at indicated positions were prepared. Sections were first blocked in 0.1M PBS containing 5% goat or donkey serum and 0.3% Triton X-100 (blocking solution) for 1 h, and then incubated with anti-PTPN13 (1:200, LS-C148268), anti-calpain-1 (1:400, LS-B4768), anti-calpain-2 (1:200, LS-C337641), Phospho-c-Abl Tyr245 (1:200, 2861, CST) and/or anti-Tau oligomer (T22, 1:300, ABN454, EMD Millipore), prepared in blocking solution overnight at 4 °C. Sections were washed 3 times in PBS (10 min each) and incubated in Alexa Fluor 488 goat anti-rabbit IgG and/or Alexa Fluor 594 goat anti-mouse IgG (1:500, Thermo Fisher Scientific) prepared in blocking solution for 2 h at room temperature. After three washes, sections were mounted with mounting medium containing DAPI (Vector Laboratories).

For analysis of PTPN13 and calpain-1/calpain-2 co-localization, sections were visualized under 100X using a confocal (Nikon) and analyzed for co-localization of the CA1 hippocampal region using ImageJ JaCOP plugin^56^ and Prism. Before analyzing using the JaCOP plugin, each image’s quality and reduction of background increased with noise despeckling, removal of bright pixel outliers with a radius of 2.0 and a threshold of 50, and with a standard increase of gamma (1.25). After which, two images of either PTPN13 and Calpain-2 or PTPN13 and Calpain-1 were compared of the same section and area of interest. Criteria looked at for the quantification of co-localization was the Pearson’s Coefficient. Image acquisition and quantification were done by two persons in a blind fashion.

For quantification of Phospho-c-Abl Tyr245 levels, 3 coronal brain sections at Bregma −0.58 mm, −1.58 mm and −1.94 mm of each brain were immunostained and analyzed. In each section, three 637 µm × 637 µm areas in both ipsilateral and contralateral sides were imaged using a confocal microscope (Nikon). Mean fluorescence intensity (MFI) in each of the three areas was measured in ImageJ and averaged. Then, MFI in the contralateral side (background signal) was subtracted from MFI in the ipsilateral side. Data in all three sections from the same brain were averaged. Image acquisition and quantification were done by two persons in a blind fashion.

For quantification of Tau oligomers, 3 coronal brain sections at Bregma −1.58 mm, −1.94 mm and −2.30 mm of each brain were immunostained and analyzed. In each section, two 424 µm × 424 µm areas in ipsilateral cortex and two in ipsilateral CA1 were imaged using a confocal microscope (LSM-880, Zeiss). Mean fluorescence intensity (MFI) in each area was measured in ImageJ. MFI_cortex_ and MFI_CA1_ in three sections from the same brain were averaged. Image acquisition and quantification were done by two persons in a blind fashion.

### ELISA

Mouse brain was homogenized in ice-cold PBS plus protease inhibitor cocktail (Thermo Fisher Scientific). 96-well ELISA plates (Nunc MaxiSorp™) were coated with 15 µg of brain homogenate using 0.05 M sodium bicarbonate, pH 9.6 as coating buffer, followed by overnight incubation at 4 °C. The plates were washed three times with Tris-buffered saline with 0.01% Tween (TBST), then blocked for 3 h at room temperature with 10% nonfat dry milk in TBST. The plates were then washed with TBST and incubated with anti-tau oligomer (1:500, T22) or anti-tau (1:2000, Tau-5, Abcam) antibody in 5% nonfat milk in TBST for 1 h at room temperature. The plates were washed three times with TBST and incubated with goat anti-rabbit IgG Horseradish-peroxidase (HRP)(1:3000, Thermo Fisher Scientific) or goat anti-mouse IgG HRP (1:3000) in 5% nonfat milk in TBST for 1 h at room temperature. Plates were then washed three times with TBST and developed with 1-Step TMB (Thermo Fisher Scientific). The reaction was stopped with 2 M sulfuric acid and the plates were read at 450 nm using a POLARstar OMEGA plate reader (BMG Labtech).

### Preparation of P2 membranes from brain homogenates

Mouse brain was isolated and placed in ice-cold homogenization buffer containing the following: 320 mM sucrose, 10 mM HEPES, pH 7.4, 2 mM EDTA and protease and phosphatase inhibitor cocktail (Thermo Fisher Scientific). Brain tissue was homogenized using a glass homogenizer. The homogenate was centrifuged at 1000 × g for 10 min, and the resultant supernatant was collected and centrifuged at 14,000 × g for 20 min. The resultant pellet (P2 membrane fraction) was centrifuged again at 14,000 × g for 20 min to eliminate protease inhibitors. P2 pellets were then resuspended in Tris-acetate buffer (100 mM Tris-acetate, pH 7.4, 50 µM EGTA) for Ca^2+^ treatment or in IP lysis/wash buffer plus protease and phosphatase inhibitor cocktail (Thermo Fisher Scientific) for immunoprecipitation.

### Immunoprecipitation

Immunoprecipitation was performed using the Pierce Classic IP Kit (26146, Thermo Fisher Scientific) following the instruction. The antibodies used for IP were PTPN13 (LS-C148268), normal rabbit IgG (2729, CST), GFP (12Ab hybridoma medium, gift from Dr. Steven Standley, Western University of Health Sciences), c-Abl (2862, CST), tau (AHB0042) and normal mouse IgG (sc-2025, Santa Cruz Biotechnology).

### Acute hippocampal slice preparation and treatment

Calpain-1 KO mice at postnatal day 10 were anesthetized with isoflurane and decapitated. Brains were quickly removed and transferred to oxygenated, ice-cold cutting medium (in mM): 124 NaCl, 26 NaHCO_3_, 10 glucose, 3 KCl, 1.25 KH_2_PO_4_, 5 MgSO_4_, and 3.4 CaCl_2_. Hippocampal transversal slices (400 μm-thick) were prepared using a McIlwain-type tissue chopper and transferred to a recovery chamber with a modified aCSF medium, containing (in mM): 124 NaCl, 2.5 KCl, 2.5 CaCl_2_, 1.5 MgSO_4_, 1.25 NaH_2_PO_4_, 24 NaHCO_3_, 10 D-glucose, and saturated with 95% O_2_/5% CO_2_ for 1 h at 37 °C. After 1 h recovery, hippocampal slices were then transferred into screw-cap microfuge tubes (2–3 slices each tube) containing 2 ml of freshly oxygenated aCSF medium with various drugs and were incubated at 37 °C. At the indicated time points, slices were rapidly frozen on dry ice. Slices were then lysed and protein concentrations were measured using BCA protein assay kit (Thermo Fisher). Equivalent amounts of proteins were processed for SDS-PAGE and western blot.

### Statistical analyses

Data are means ± SEM in all figures. To compute p values, unpaired two-tailed Student’s t-tests and one- or two-way ANOVA with Bonferroni post-tests were used, as indicated in figure legends.

### Data availability

The datasets generated during and/or analyzed during the current study are available from the corresponding author upon reasonable request.

## Electronic supplementary material

Supplementary Data
