# Supplementary material for: The tyrosine phosphatase PTPN13/FAP-1 links calpain-2, TBI and tau tyrosine phosphorylation
Source: Sci Rep. 2017 Sep 18;7:11771. doi: 10.1038/s41598-017-12236-3 (PMC5603515; doi:10.1038/s41598-017-12236-3)
Supplement: Supplementary file 1 — Supplementary Data [file 41598_2017_12236_MOESM1_ESM.doc]

**The tyrosine phosphatase PTPN13/FAP-1 links calpain-2, TBI and tau tyrosine phosphorylation**

Yubin Wang^1^, Randy A. Hall^3^, Moses Lee^1^, Andysheh Kamgar-parsi^1^, Xiaoning Bi^2^ and Michel Baudry^1^

^1^Graduate College of Biomedical Sciences

^2^College of Osteopathic Medicine of the Pacific

Western University of Health Sciences

Pomona, CA 91766

and

^3^Emory University School of Medicine

Atlanta, GA  30322

**Running title:** Calpain-2, PTPN13 and tau phosphorylation

Send Proofs and Correspondence to: Dr. Michel Baudry

Western University of Health Sciences

309 E. 2^nd^ St

Pomona, CA 91766

Email: [mbaudry@westernu.edu](mailto:mbaudry@westernu.edu)

Tel: 909-469-8271


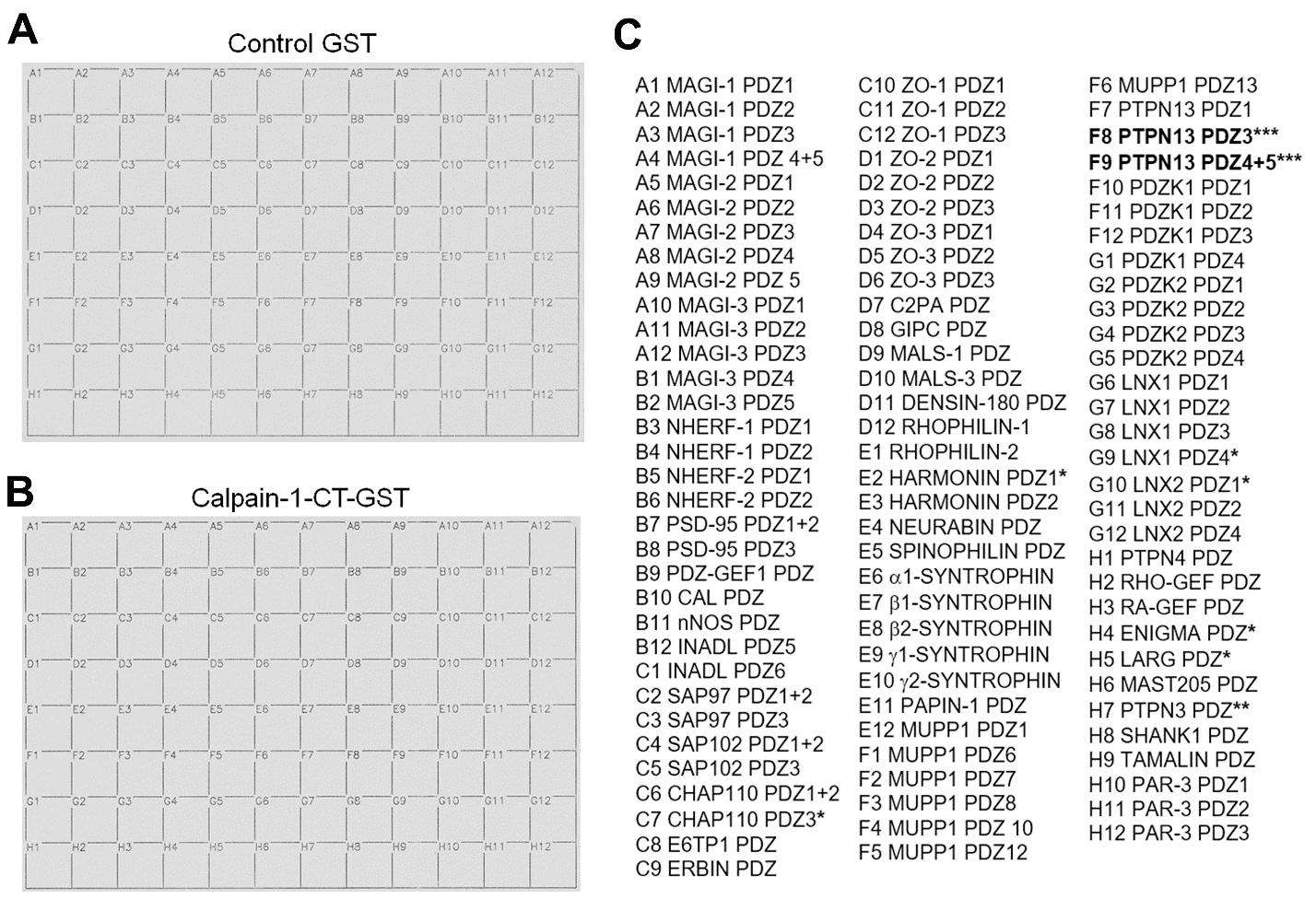


**Figure S1.** A screening of PDZ domains interacting with GST-tagged calpain-1 C-terminus.

**A, B.** The screening of PDZ domains interacting with the GST (A) or GST-tagged calpain-1 C-terminus (B) in a type I PDZ binding domain array.

**C.** The list of PDZ domains in the type I PDZ domain array used in Fig. S1A, B and in Fig. 1A. *, ** or *** indicates the binding strength with calpain-2 C-terminus.


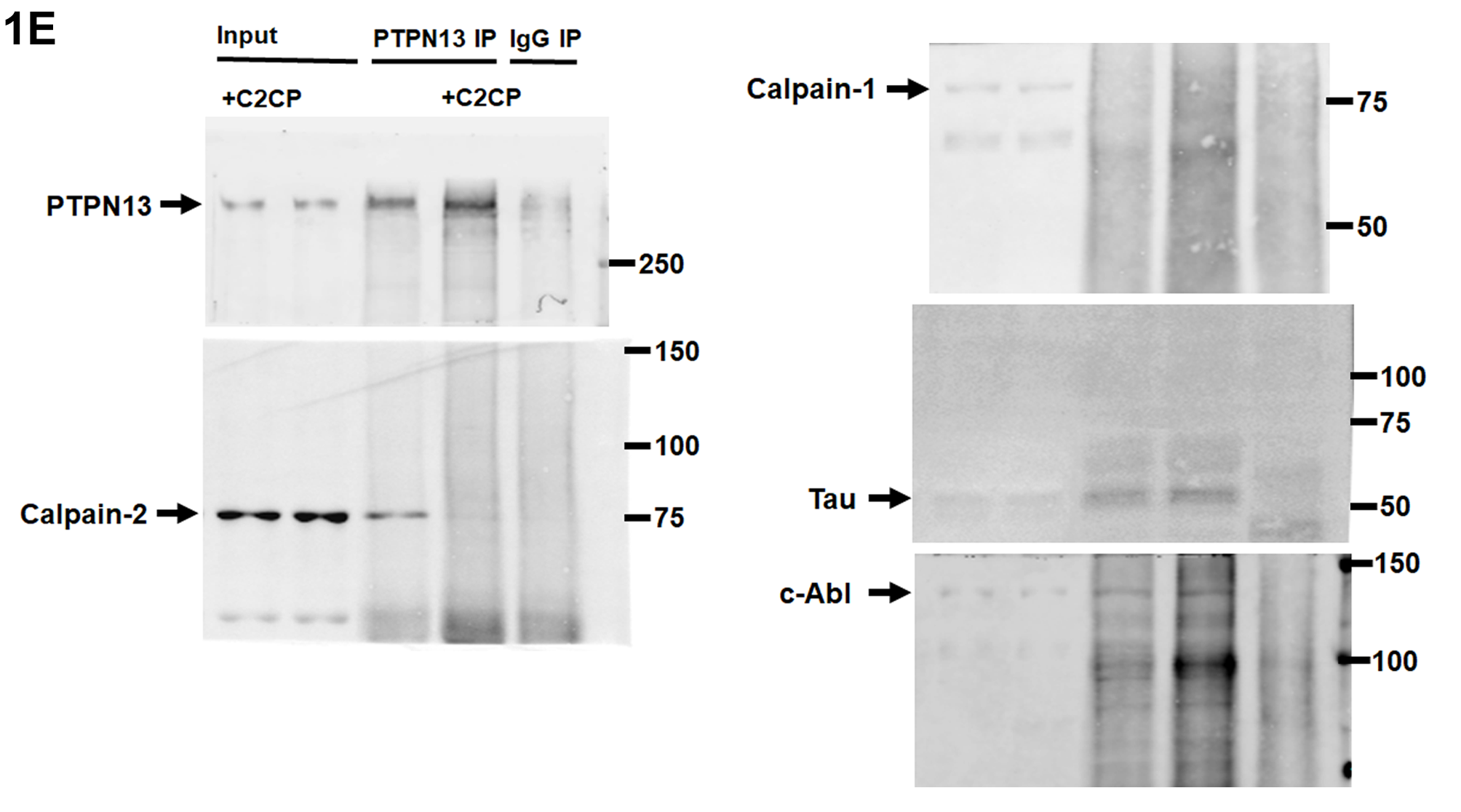

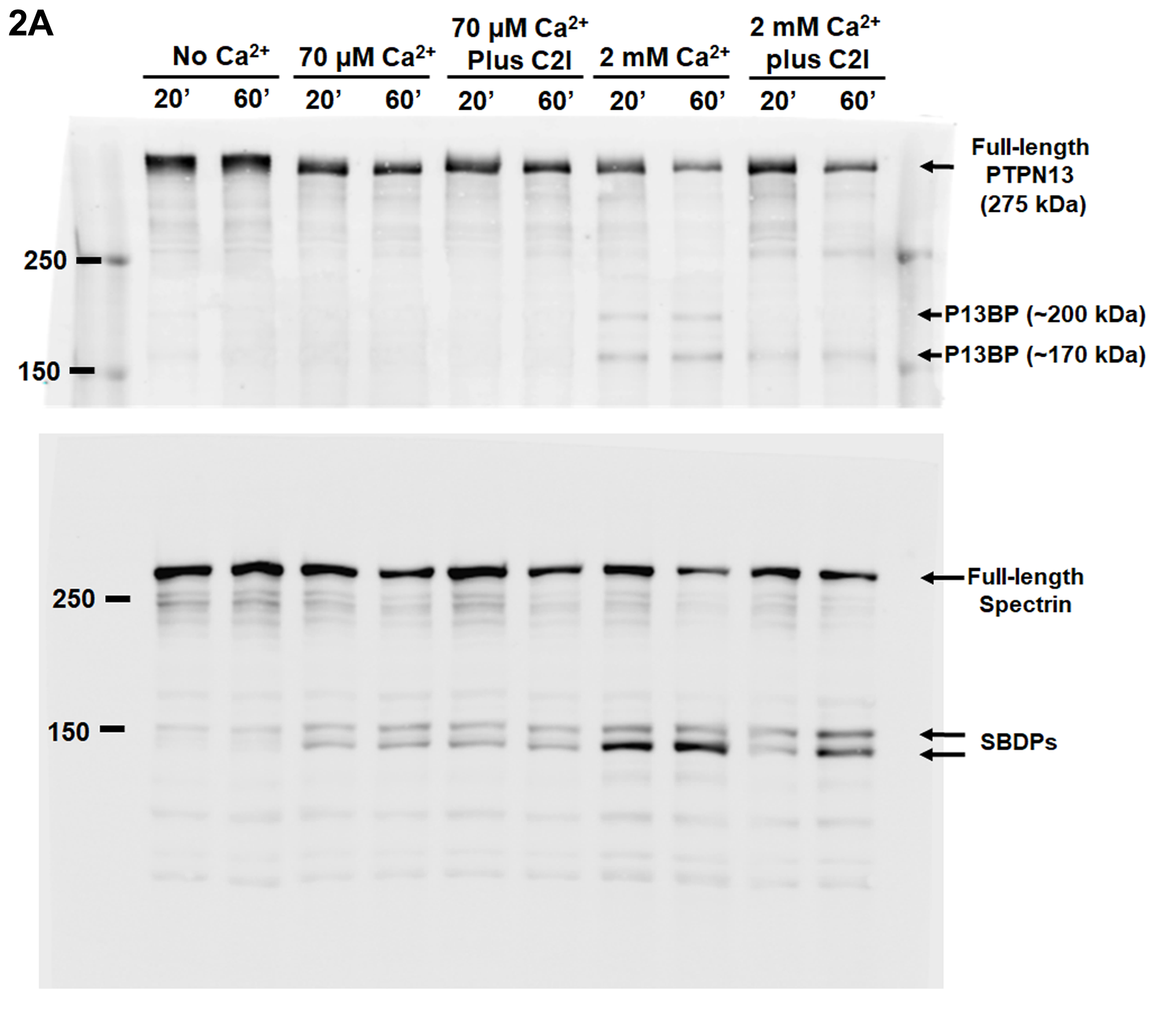


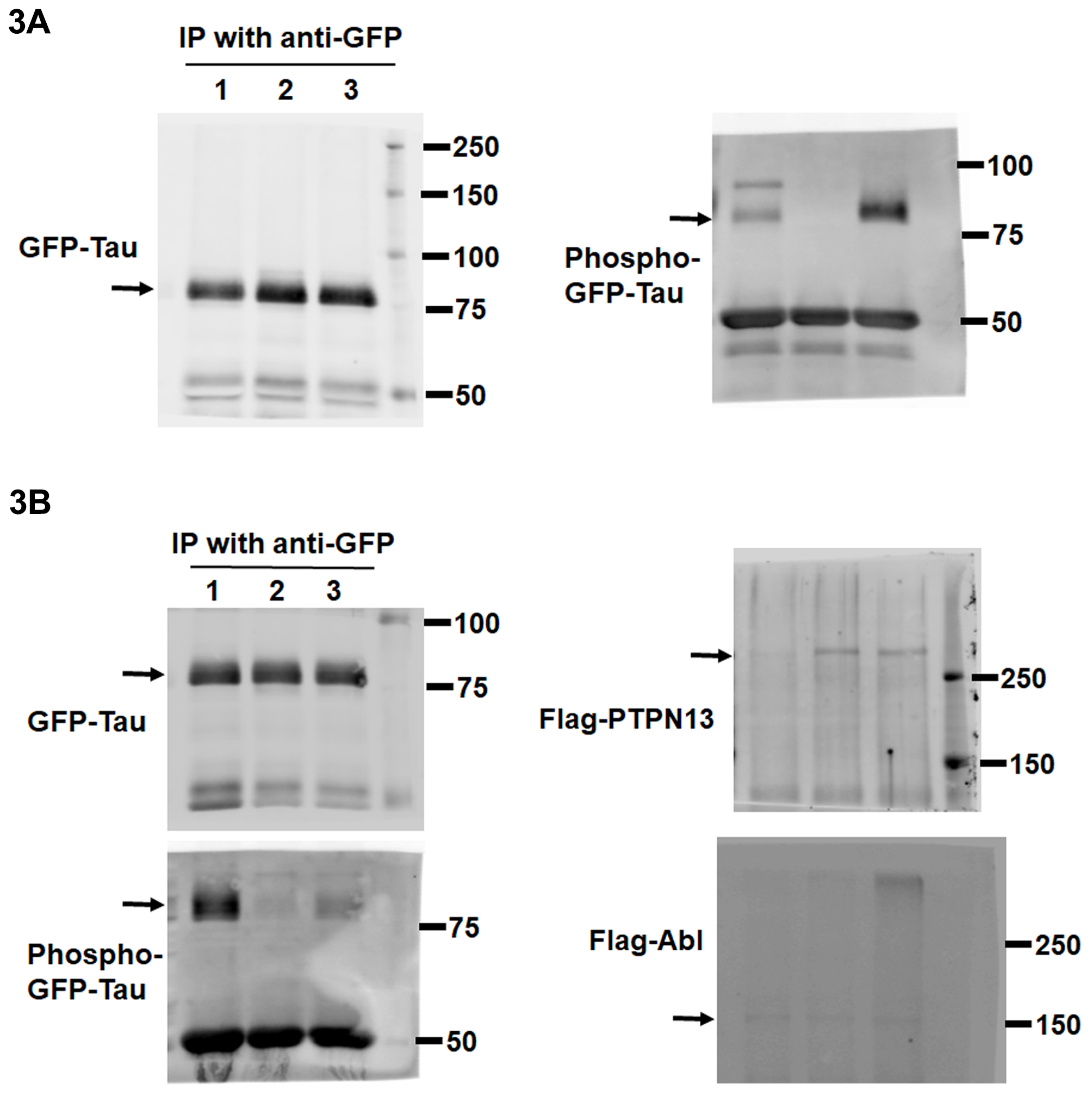


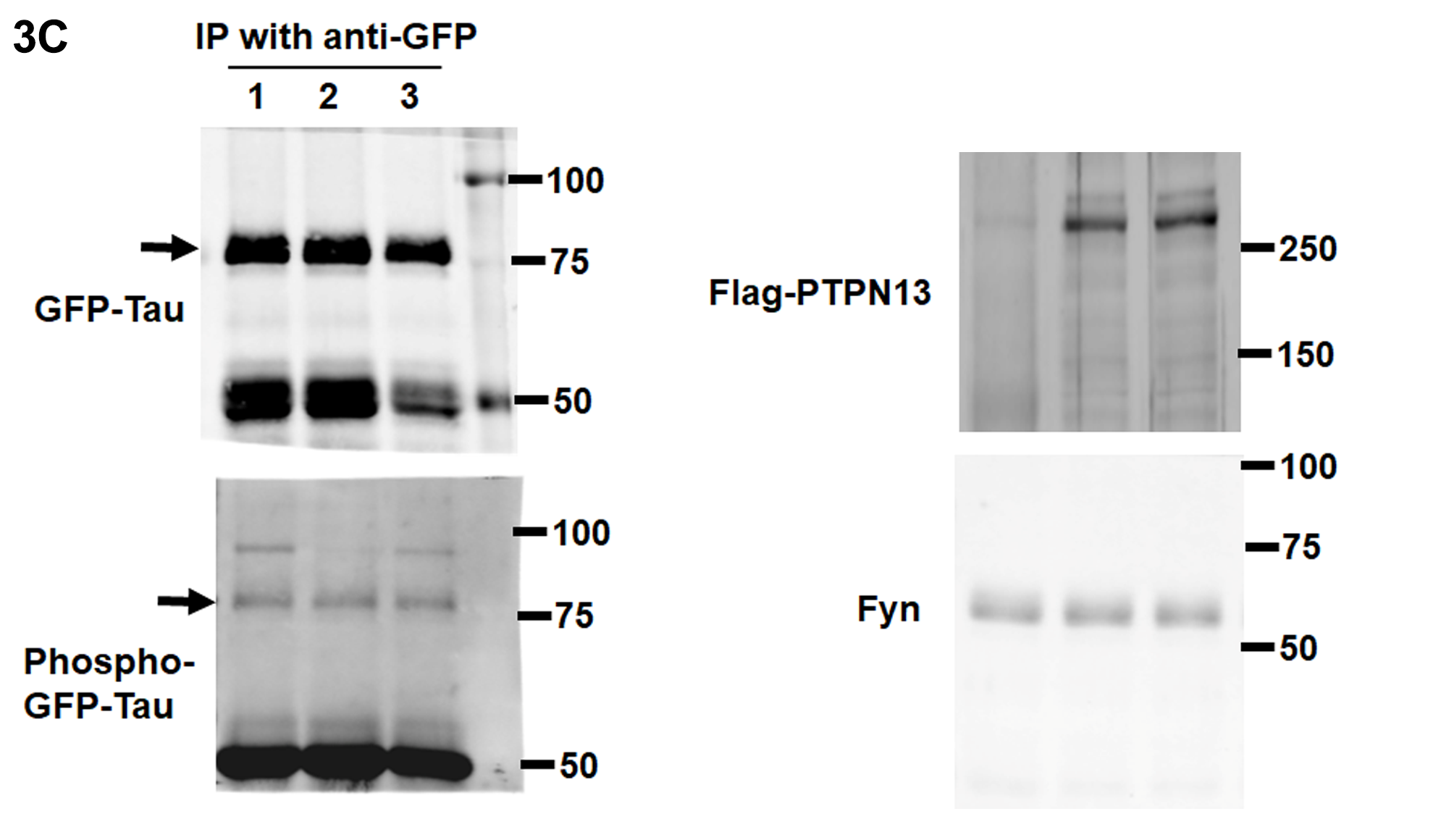


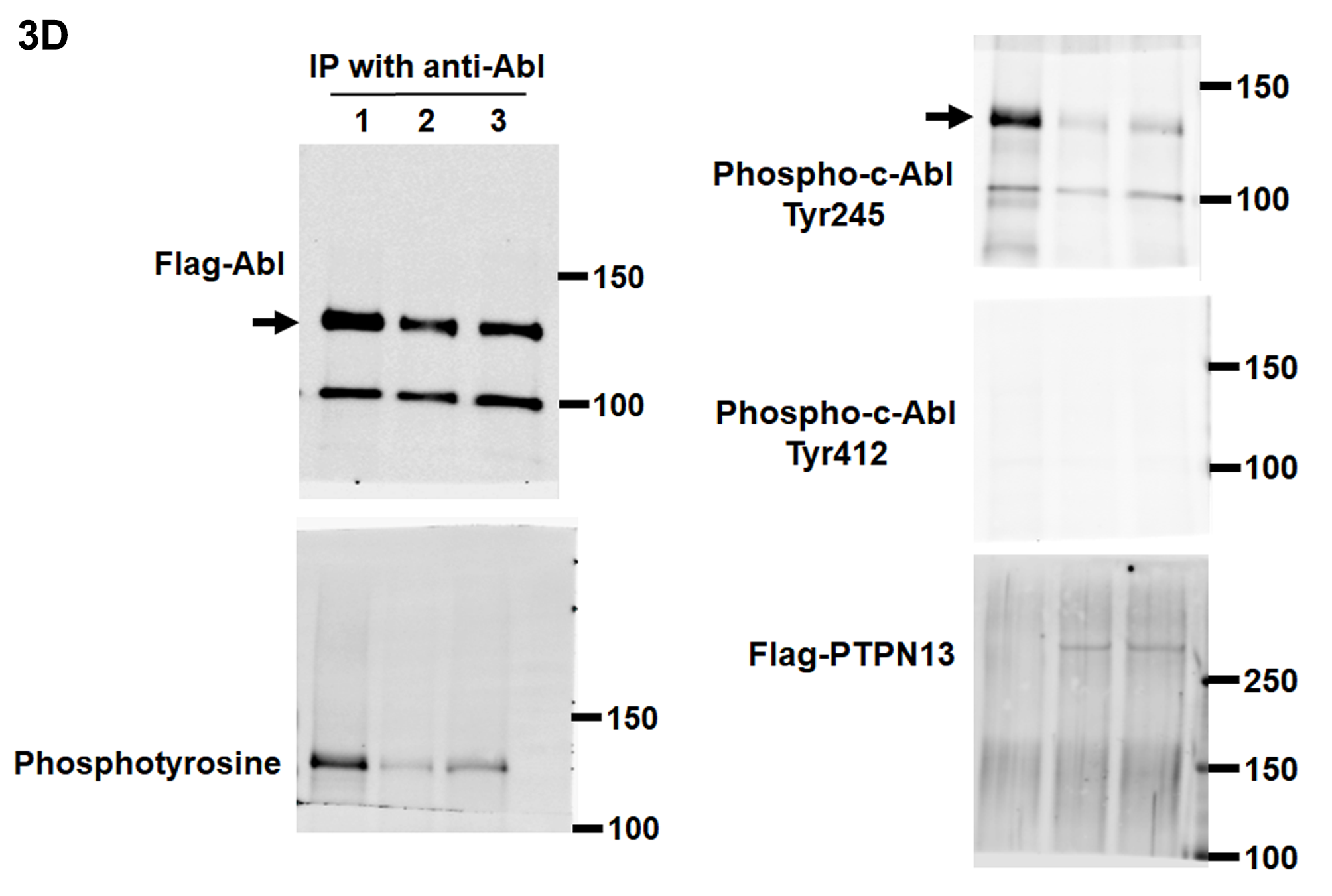


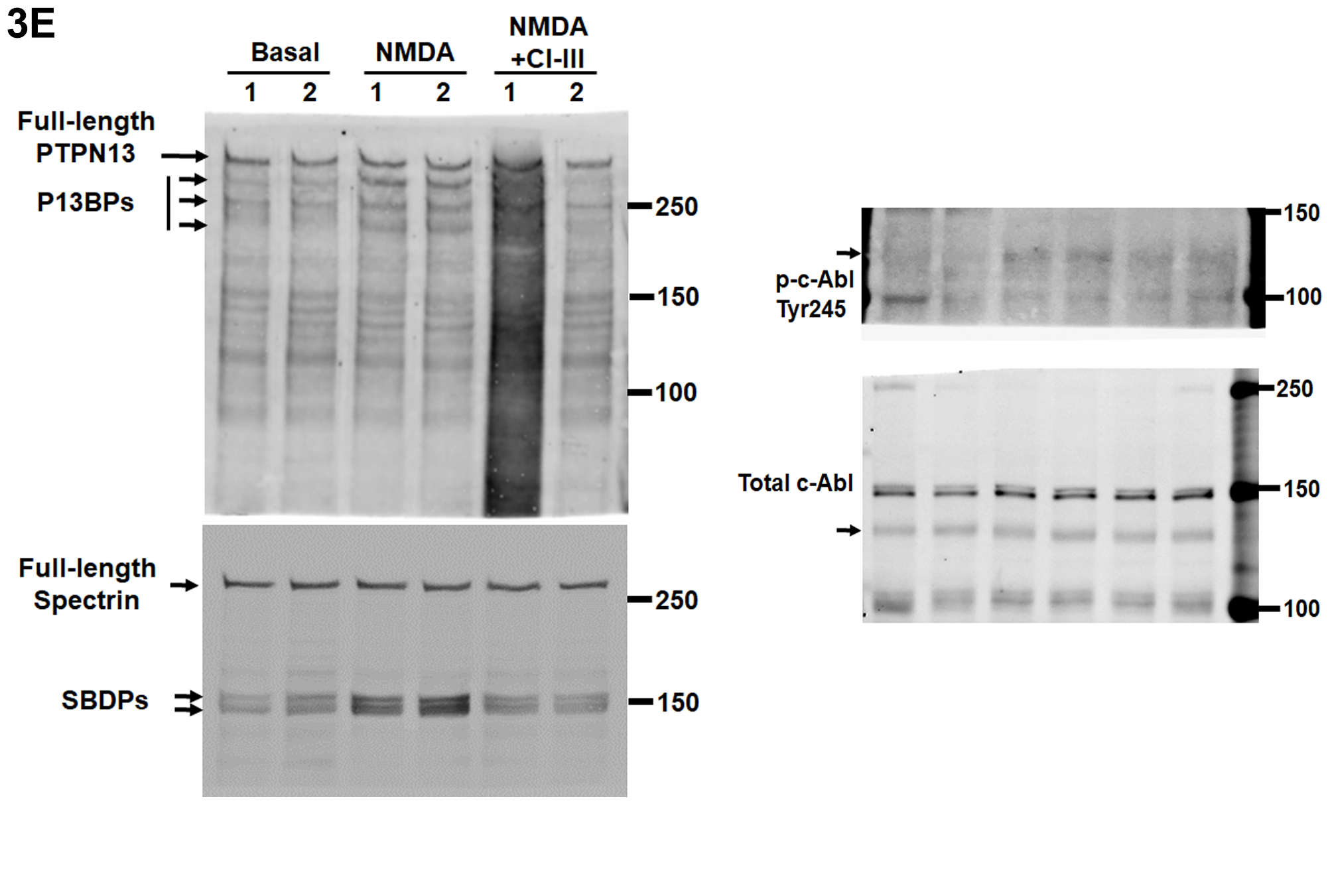


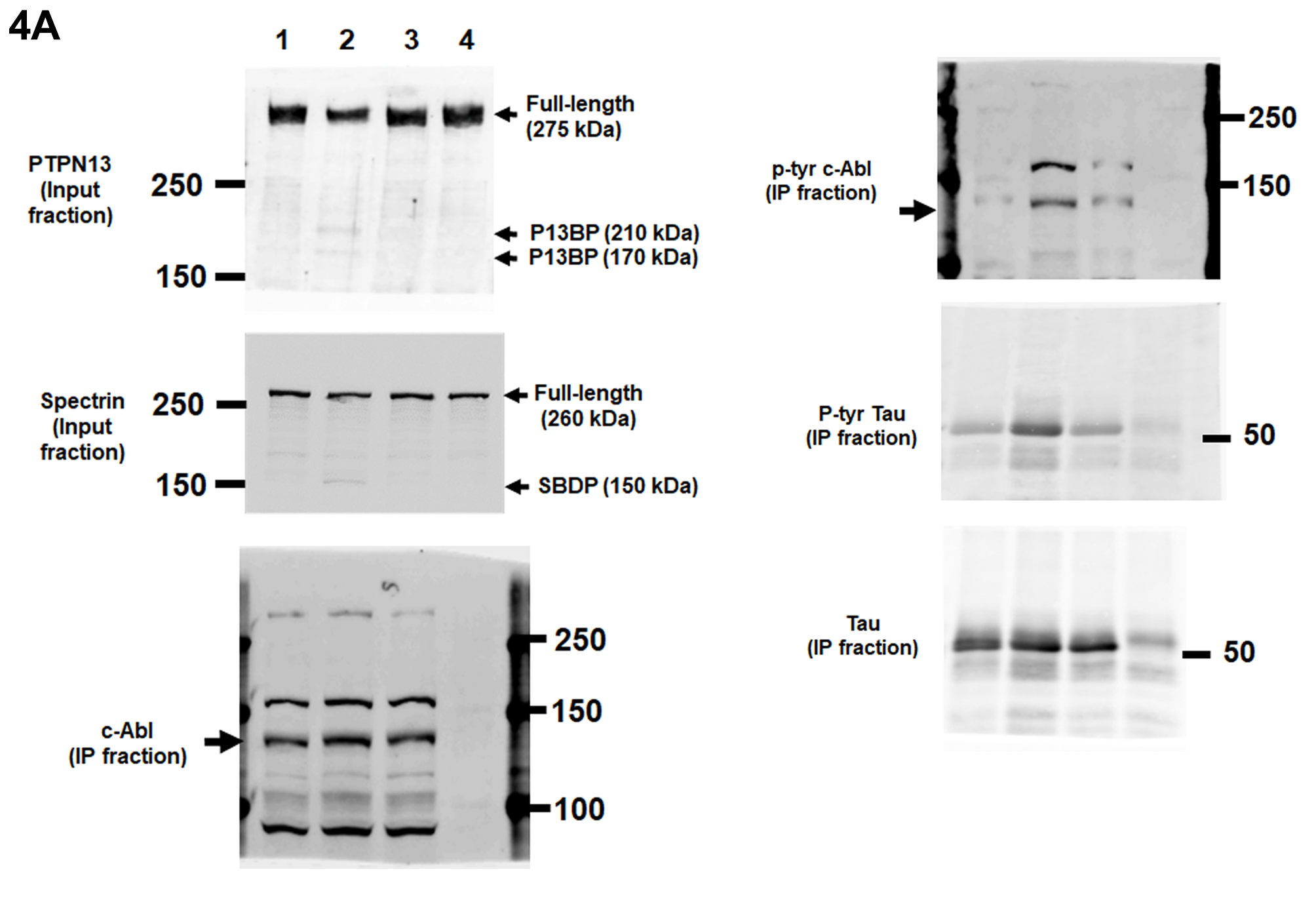


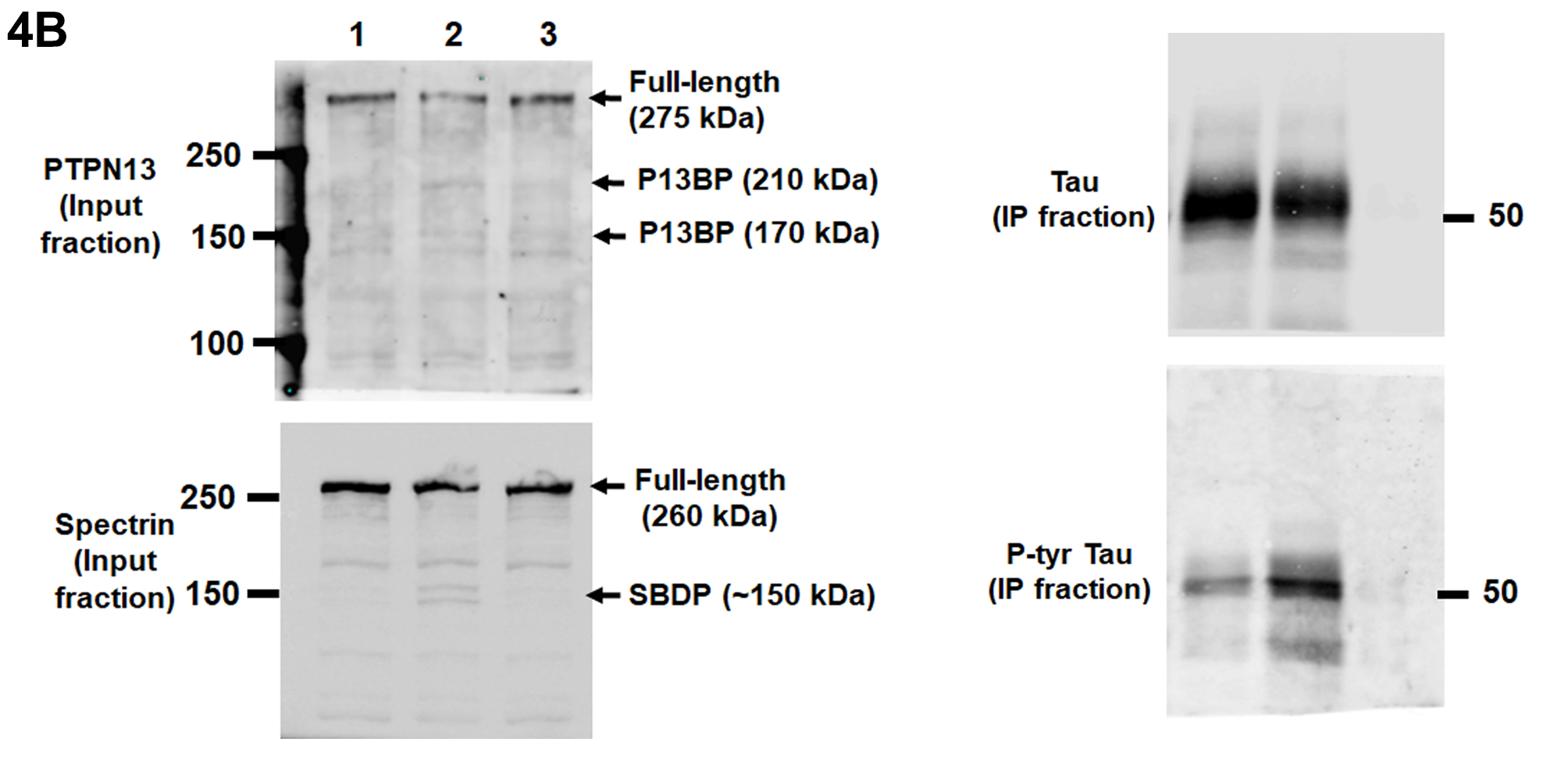


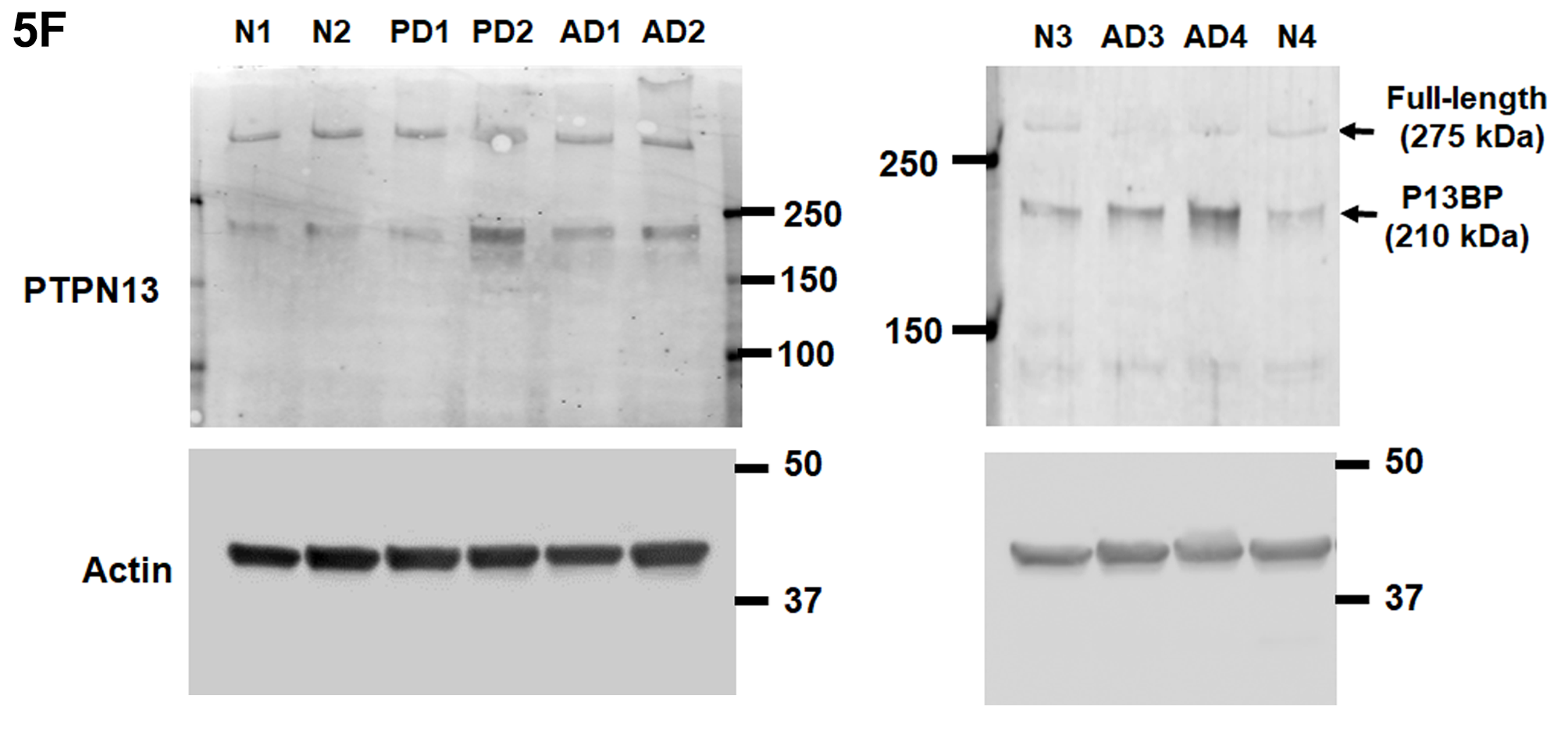


**Figure S2.** Full-length images of the cropped blots in the main figures.
